# Can the High Sensitivity of Xpert MTB/RIF Ultra Be Harnessed to Save Cartridge Costs? Results from a Pooled Sputum Evaluation in Cambodia

**DOI:** 10.3390/tropicalmed5010027

**Published:** 2020-02-15

**Authors:** Monyrath Chry, Marina Smelyanskaya, Mom Ky, Andrew J Codlin, Danielle Cazabon, Mao Tan Eang, Jacob Creswell

**Affiliations:** 1 Cambodia Anti-Tuberculosis Association, Phnom Penh and Cambodia, Phnom Penh 12250, Cambodia; rath@thecata.org.kh (M.C.); momky@thecata.org.kh (M.K.); 2 Stop TB Partnership, TB REACH, 1218 Geneva, Switzerland; marinas@stoptb.org (M.S.); andrew.codlin@gmail.com (A.J.C.); 3 McGill International TB Centre, McGill University, Montreal, QC H4A 3J1, Canada; danielle.cazabon@gmail.com; 4 National Center for Tuberculosis and Leprosy Control (CENAT), Phnom Penh 12250, Cambodia; mao@online.com.kh

**Keywords:** TB diagnostics, laboratory methods, case detection, Xpert Ultra

## Abstract

Despite the World Health Organization recommending the use of rapid molecular tests for diagnosing tuberculosis (TB), uptake has been limited, partially due to high cartridge costs. Other infectious disease programs pool specimens to save on diagnostic test costs. We tested a sputum pooling strategy as part of a TB case finding program using Xpert MTB/RIF Ultra (Ultra). All persons were tested with Ultra individually, and their remaining specimens were also grouped with 3–4 samples for testing in a pooled sample. Individual and pooled testing results were compared to see if people with TB would have been missed when using pooling. We assessed the potential cost and time savings which different pooling strategies could achieve. We tested 584 individual samples and also grouped them in 153 pools for testing separately. Individual testing identified 91 (15.6%) people with positive Ultra results. One hundred percent of individual positive results were also found to be positive by the pooling strategy. Pooling would have saved 27% of cartridge and processing time. Our results are the first to use Ultra in a pooled approach for TB, and demonstrate feasibility in field conditions. Pooling did not miss any TB cases and can save time and money. The impact of pooling is only realized when yield is low.

## 1. Introduction

Early and increased diagnosis of tuberculosis (TB) remains a key part of the World Health Organization’s (WHO) End TB Strategy [1]. Almost a decade ago, the introduction of the Xpert MTB/RIF (Xpert) assay presented an opportunity to transform the ways in which TB is diagnosed and care is delivered. The Xpert assay can rapidly detect resistance to rifampicin and has demonstrated superior sensitivity in diagnosing pulmonary [2], extrapulmonary [3], and pediatric [4] TB when compared to its century-old predecessor—acid fast bacilli (AFB) smear microscopy. Xpert testing has led to an increase in multidrug-resistant (MDR) TB detection [5] and an increase in bacteriological confirmation of drug-sensitive TB where it has been scaled up [6,7]. Xpert was initially only recommended as the initial test for TB in people living with HIV and those at increased risk of MDR-TB [8]. However, the WHO now recommends that Xpert be used as a first-line test for everyone with presumptive TB [9]. Yet, in many settings, especially in countries with a high burden of TB, AFB smear microscopy remains the mainstay of TB testing despite missing 40%–50% of people with TB [10,11].

The high cost of installing and operationalizing a GeneXpert testing network is one of the reasons for the continued low levels of Xpert coverage in many countries. Despite an over 40% reduction in Xpert cartridge costs [12], the current USD 9.98 price per cartridge is still high when compared to AFB smear microscopy [13]. The impact of Xpert’s higher cost is particularly acute in high TB burden countries, most of which are still highly dependent on international donor resources to fund their TB response [14]. The Global Fund to Fight AIDS, TB, and Malaria (Global Fund) is currently the largest donor for TB internationally, and has invested significantly in the procurement of GeneXpert systems and Xpert cartridges [14]. However, even with the Global Fund’s substantial support for Xpert cartridges and machine procurement, the resources needed to test all people with presumptive TB using a rapid molecular test at current prices are well beyond available funding levels.

To reduce spending on diagnostic testing, other infectious disease programs have utilized a pooled testing strategy. Pooling involves combining several specimens of blood, urine, sputum etc. into a common pool which is then tested. If the pooled test result is negative, all of the individual specimens are declared as negative without being individually retested. If the pooled test result is positive, at least one of the pool’s component specimens is positive, and all of the component specimens should be individually retested to identify which ones are positive and negative. This testing strategy has been used for screening in blood banks [15] and sexually transmitted infections including HIV [16,17,18]. While a pooled sputum specimen strategy has the potential to bring about significant cost savings, the technique has not been readily adopted as a TB testing strategy. However, the few studies which have been published on this approach show its promise. A study evaluating the pooled Xpert testing strategy in Nigeria showed that it would have identified 96% of individual specimens containing TB bacteria and could have reduced Xpert costs and usage by 30% [19]. Another evaluation in South Africa showed that Xpert identified 100% of pools containing AFB smear-positive specimens, but just 76% of pools with AFB smear-negative, culture-positive specimens [20].

One major concern with the testing of pooled sputum specimens from multiple people with presumptive TB is that TB bacteria may be diluted to a concentration which is below the detection limits of the Xpert assay, thus producing false negative results. Sputum pooling should be more feasible if the sensitivity of the test is very high, i.e., can identify disease even in diluted samples. In 2018, Cepheid released its next-generation Xpert test—the Xpert MTB/RIF Ultra (Ultra) assay. Initial reports indicate that the Ultra assay can improve sensitivity compared with Xpert. The Ultra assay improved sensitivity by 5% among all people enrolled. However, among groups with lower bacterial burdens such as individuals with smear-negative, culture-positive results and people with HIV infection, the gains in sensitivity were much higher (17% and 12% respectively) [21].

Cambodia is a high TB burden country, and only an estimated 58% of people with TB in the country are diagnosed and have access to treatment [14]. The 2011 Cambodia TB prevalence survey identified that the largest gaps are in diagnosing patients over 55 years of age, and highlighted that many people with TB presented no symptoms [22]. In addition, in rural areas access to TB testing and specifically molecular testing is limited. Our intervention took this into consideration, focusing on people aged 55 years and older, providing chest X-ray (CXR) screening in addition to a symptom screen, and bringing a mobile lab to rural areas to test with Ultra. Results presented in this paper evaluate the concordance of test results and potential cost implications of a pooling strategy embedded within an active case finding intervention in rural Cambodia using Ultra.

## 2. Methods

### 2.1. Active Case Finding Setting

This evaluation of a pooled testing strategy was embedded within an active TB case finding (ACF) initiative in Cambodia funded by the Stop TB Partnership’s TB REACH initiative. The Cambodia Anti-Tuberculosis Association (CATA) organized a series of mobile CXR screening days in rural operational districts (ODs) from 12 June 2017 to 16 May 2018. The TB screening activities were aimed at individuals aged 55 years and older, similar to what has been described elsewhere [23]. In addition to engaging older individuals, screenings also targeted other villagers who reported cough and/or other TB symptoms. Village Health Support Groups (VHSG) conducted door to door outreach and sensitization activities prior to testing days and referred older individuals and people presenting with TB symptoms and aged under 55 to local health centers. On testing days, the mobile unit equipped with an X-ray machine and a 4-module GeneXpert, was parked at the local health center to facilitate testing. As part of the larger ACF initiative, we pooled samples using Ultra on a subset of individuals as the Ultra assay was in limited supply and we wanted to evaluate the feasibility of using a pooled method in future ACF events. On certain days, the team would agree to use Ultra cartridges and pool the samples. During the other screening days, the ACF initiative proceeded as described.

All older individuals (55 years and over) attending the screening day received a CXR and those under 55 years of age were verbally screened and eligible for CXR if they reported a current cough of any duration. CXR results were read by a trained radiologist and classified into four categories: (1) active TB; (2) presumptive TB/suspected TB abnormality; (3) healed TB/other lung abnormality; (4) CXR normal. Any individual with an abnormal CXR and people with a reported cough were eligible for sputum collection, so CXR was not used to screen people out of the testing algorithm but rather for classification.

### 2.2. Pooling of Specimens and Ultra Testing

Sputum specimens were individually tested using the Ultra assay according to the manufacturer’s instructions with a 1:2 sputum to reagent ratio. Individuals with an error, invalid, or no result in the Ultra test outcome were retested to obtain a valid positive or negative result for the detection of *Mycobacterium tuberculosis* (MTB). The individual result was used as the definitive result for this analysis and for treatment decisions. Anyone with an MTB-positive result including trace call results on the individual Ultra test were eligible for appropriate TB treatment according to the National Center for Tuberculosis and Leprosy Control (CENAT) guidelines.

Remnant specimens, a mixture of sputum, and Ultra Sample Reagent which was left over after individual Ultra testing were grouped into batches of three or four containers. We attempted to group remnant specimens from individuals by their CXR classification so that a pooled specimen would contain remnant specimens from all CXR abnormal or all CXR normal individuals. However, this was not always possible due to delays with CXR reading and variance in the types of participants who were mobilized and tested each day. Thus, a small subset of remnant specimen groupings contained a mix of individuals with CXR abnormal and CXR normal results. At the testing site, we took 0.75mL of each individual specimen (to ensure that 2.25–3mL of total specimen was available for testing) which was deposited into a new sputum container and swirled inside the container for 5–10 seconds to mix the sample thoroughly. The pooled specimen was then tested using the Ultra assay according to the manufacturer’s instructions.

### 2.3. Data Management and Analysis

Individual and pooled Ultra test results were entered into a database using the coding scheme recommended by the Global Laboratory Initiative [24]. The data were cleaned, checked for inconsistencies, and analyzed using R version 3.5.1 (R Foundation for Statistical Computing, Vienna, Austria). We calculated the agreement between the individual Ultra results and the pooled Ultra results for detecting MTB, as well as rifampicin (RIF) resistance. We calculated the theoretical cost and time savings that could be achieved by using a pooling strategy with different approaches. For the analysis, the cost of an Ultra cartridge was estimated to be USD 10.36 (including shipping and import fees) based on shipping information from the Global Drug Facility at Stop TB Partnership, and we used a 90 minute runtime for the Ultra assay [25].

### 2.4. Ethical Approval

Approval to conduct this programmatic intervention was obtained from the National Center for Tuberculosis and Leprosy Control CENAT. Verbal consent was obtained; individuals were able to refuse participation at any point during the screening, diagnosis, or treatment process without compromising their access to the routine care provided by CENAT. De-identified data were used for this analysis.

## 3. Results

### 3.1. Individual Test Results

A total of 584 individual Ultra results were analyzed. These individuals were 50.5% (n = 295) female and 49.5% (n = 289) male; 89% (n = 522) were aged 55 and above. Basic demographic, screening, and TB risk characteristics are presented in Table 1. In total, 91 individual samples (15.6%) had positive results on Ultra. These included 3 (3.3%) with rifampicin resistance, 1 (1.1%) whose rifampicin result was indeterminate, and 13 (14.3%) individuals who had trace calls (see Table 2). Of the 13 people with individual trace call results, two had previous treatment history and 10 had CXRs that were read as ‘active TB’ or ‘presumptive TB’.

A total of 227 individuals had a CXR reading of “active TB” or “presumptive TB” and 73 (32%) out of this subgroup were identified as being MTB-positive. By contrast, among 301 samples from individuals with normal CXR readings, only 2 were identified as being MTB-positive. Overall, samples from pools with CXR abnormalities (active, presumptive, or healed TB) contributed 78 out of 91 (85.7%) of all the MTB-positive cases (Table 3).

### 3.2. Pooled Results

There were in total 153 pooled samples that were tested. There were 28 pools with three individual samples and 125 pools containing four individual samples. Of the 153 pools, 66 were made from samples from individuals with abnormal CXR readings (“active TB”, “healed TB”, “presumptive TB”, “other CXR abnormality”), 70 were made from samples from individuals with normal CXR readings, and 17 were mixed (any CXR abnormality and CXR normal reading). Among the 153 pooled samples, 73 (47.7%) had an MTB-positive result and 80 were negative on Ultra. Of the 80 negative pools, 68 (85%) came from CXR normal sample pools and the other 12 came from CXR abnormal or mixed pools. All the 304 individual test results from the 80 negative pools tested negative, indicating 100% agreement, and no extra testing would have resulted in more MTB-positive individuals being detected. Table 2 summarizes the pooled and individual testing results.

All 73 pools with an MTB-positive result had at least one MTB-positive individual test result, indicating that no false positive results were found in the pooled samples. Seventeen (23%) of the 73 positive pools contained multiple individual samples that tested for MTB-positive; 16 pools had two positive individual samples, one pool had three individual positive samples, and the remaining 56 had one individual positive sample. Out of the 73 positive pools, 60 (82.2%) came from pools composed of CXR abnormal samples, 11(15.1%) came from pools composed of mixed abnormal and normal samples, and 2 (2.7%) came from pools containing only CXR normal samples.

Of these 73 pooled samples with MTB-positive results, 0 had rifampicin-resistant results, 3 (5.4%) had rifampicin indeterminate results, and 11 (15%) were trace calls (Table 2). The three pools with MTB-positive results with no rifampicin resistance detected, revealed rifampicin resistance in the individual sample during individual testing. These rifampicin-resistant samples would not have been missed, since all the positive pools were retested. The 11 pooled samples with trace call results contained 44 individual results of which 12 were positive. Among the 12 positive individuals, seven samples were MTB-positive, four had trace calls (two of these came from the same pool), and one was MTB-positive with indeterminate rifampicin resistance.

### 3.3. Cartridge Savings and Impact of CXR Screening on Costs

If this ACF initiative had only conducted direct, individual Ultra testing, it would have used 584 Ultra cartridges, requiring 876 h of testing run times and costing a total of USD 6050 for cartridge procurement (Table 4). Our pooled testing approach used 153 Ultra cartridges to test pooled specimens (229 h of testing run times and a cost of USD 1585). An additional 280 cartridges would have been required for all of the individual specimens from the 73 pooled specimens with positive results (420 h of testing run times and a cost of USD 2900). In total, 433 Ultra cartridges would have been used representing a 26.8% reduction in Ultra cartridge costs and testing workloads with no loss of yield.

Since the pooled specimens containing only individual samples from people with abnormal CXR results had such a high positivity rate (90.9%), the testing strategy which results in the largest reductions in costs and testing workloads is actually a hybrid strategy, where individuals with an abnormal CXR have their individual sputum specimens directly tested and where the sputum specimens from people with normal CXR results are pooled before testing. If this hybrid strategy had been used, we would have directly tested 304 specimens from people with abnormal results, 70 pooled specimens from people with normal CXR results, and just 8 individual specimens after a positive pooled test result. This testing strategy would have required a total of 382 Ultra cartridges (573 h of testing run time and USD 3958 in cartridge costs representing a 34.6% reduction in Ultra cartridge costs and testing workloads compared to direct, individual Ultra testing).

## 4. Discussion

Our study adds to a very small but intriguing body of literature on the possible benefits of pooling sputum specimens for testing with molecular assays. This is the first published account of pooling with the Ultra assay. Two previous studies from Nigeria [19] and South Africa [20] used the Xpert MTB/RIF assay, and while both of these studies document efficiency gains in terms of workloads and cost, they both also reported missing a small number of people with TB in the pooled specimens (only 4% in Nigeria, but pooling missed 24% of smear-negative, culture-positive samples in the South African study). By contrast, our pooled testing evaluation with the Ultra assay showed that no pooled specimens with a negative result contained individual MTB-positive samples.

The detection limit of the Ultra assay is much lower than Xpert MTB/RIF, which should reduce the risk that pooling dilutes TB DNA below the detection limits of the molecular assay [21]. A controlled laboratory study using spiked sputum demonstrated the Xpert MTB/RIF cycle threshold increased with the dilution of TB DNA, but still managed to detect the majority of MTB-positive specimens [26]. It would logically follow that the use of Ultra would improve these results and that pooling with Ultra may advance the access of more individuals to rapid TB testing.

Pooling has been successfully used in other infectious diseases, but the premise of pooling is only useful when testing yields are low. Our study demonstrates that if the pre-test probability of a positive result is high then pooling multiple specimens together only increases the likelihood that the pooled result will be positive, resulting in more instead of less testing. For example, at an MTB-positive prevalence of 16%, the probability of having a pooled test result combining four individuals which contains at least one positive individual specimen is roughly 50%, and at 10% prevalence, this probability drops to 34%. When MTB-positive prevalence is 5%, more than 80% of pooled specimens will contain all negative individual specimens, offering high rates of expected savings. A laboratory-controlled study on pooling samples found good results of Xpert when pooling up to 11 negative samples with one spiked positive sample, but the authors noted the logistical challenges of using more than four or five samples for each pool [26].

The relationship between the pre-test probability and potential cartridge and time savings is then modified by the use of other screening tools, such as CXR. Because the interpretation of CXR findings can greatly increase the pre-test probability of having MTB-positive results [27], programs may want to stratify the results of the CXR to maximize cost savings. In our study, the potential cost and cartridge savings of a universal pooling strategy was 26%, but even greater efficiencies could have been achieved by pooling only CXR normal samples, which would have saved 35% of costs and cartridges. Implementing a pooled testing approach would have been more costly if used among those with a CXR reading of active TB because so many pools would have required retesting. While traditional CXR equipment is expensive and challenging to take on the road, the advent of digital CXR with computer-aided reading (CAR) has the potential to reach wider population groups and be less costly over time. The ability of CAR to provide continuous probability scores gives TB screening and testing programs an opportunity to develop cutoff scores to maximize the cost savings for pooling based on the artificial intelligence scoring [28].

The main concern regarding the use of Ultra has been its loss of specificity when testing samples from individuals with previous history of TB treatment [21]. In our sample of 584 individuals, 11 samples demonstrated the lowest bacillary burden also identified as “trace call”. Of these 11, only two individuals had been previously treated. These two had symptoms and CXR readings that suggested TB, so the decision was made to treat both individuals for TB. The other nine individuals had symptoms and suggestive CXR readings and were also treated for TB. More research and presentation of results on trace calls need to be published and considered to help programs understand the implications of trace call results. We were not able to conduct culture as part of the study nor do we have data on the cycle thresholds, which could potentially provide more insight into the trace call results as well as support the individual testing results, which is a limitation.

Our study accompanied an active TB screening and testing campaign, and we thus had access to a large quantity of samples and could test them almost immediately after obtaining them. We also had experienced lab technicians working on labeling and testing the samples who received specific training on the pooling protocols, and had access to an adequate number of GeneXpert systems. While the study was done in field conditions, certain preparations were made to ensure rapid testing of the samples and adequate recording of pooled and individual test results, including additional oversight from the research team, special templates for grouping samples and documenting results, and end of the day reports. Our time savings analysis does not take into account the extra time it takes to prepare a pooled sample, but this time was not considered substantial. Attempts at pooling specimens to increase laboratory throughput may consider such challenges as long-term storage of samples, inconsistencies in recording, contamination, and staff shortages. Thus, if the practice is to be adopted, further training and refining of procedures have to be developed. Our results are promising regarding the feasibility of pooling samples with Ultra in field conditions. Programs can decide to attempt to use pooling based on the expected or observed yield in the tested population.

## 5. Conclusions

Our results demonstrate that pooling specimens from multiple individuals with presumptive TB for testing with the Ultra assay is feasible in field conditions. Individual test results showed that no one with TB nor rifampicin resistance would have been missed by employing a pooled testing approach. TB screening and testing campaigns testing a large number of individuals with an expected low yield can pool sputum samples to save both cartridge costs and time. Additional research is needed to document results across different settings to ensure that similar results are obtainable, and that pooling use can be used at a larger scale.

## Figures and Tables

**Table 1:** Demographic information for individuals tested with Xpert MTB/RIF Ultra (Ultra).

Individuals Tested with Ultra	n = 584	%	Individuals with Positive Ultra Results	n = 91	%
***Sex***			***Sex***		
Female	295	50.5%	Female	31	34%
Male	289	49.5%	Male	60	66%
***Age***			***Age***		
<35	9	1.5%	<35	2	2.2%
35<55	53	9.1%	35<55	16	17.6%
>55	522	89%	>55	71	78.0%
***Employment Status***			***Employment Status***		
Elderly/retired	78	13.4%	Elderly/retired	13	14.3%
Factory worker	2	0.3%	Farmer	55	60.4%
Farmer	413	70.7%	Government officer	2	2.2%
Government officer	9	1.5%	Unemployed	12	13.2%
Unemployed	59	10.1%	Own business	4	4.4%
Own Business	10	1.7%	Private company staff	1	1.1%
Private company staff	1	0.2%	Seller	4	4.4%
Seller	12	2.1%			
***Symptom screen***			***Symptom Screen***		
Any symptom	556	95.2%	Any symptom	86	94%
No symptoms	28	4.8%	No symptoms	5	5.5%
Cough alone	43	7.7%	Cough alone	5	5.5%
***Chest X-ray (CXR) Results***			***CXR Results***		
CXR active	115	19.7%	CXR active	47	51.6%
CXR presumptive Tuberculosis (TB)	112	19.2%	CXR presumptive TB	29	31.9%
CXR healed	45	7.7%	CXR healed TB	13	14.3%
CXR normal	301	51.5%	CXR normal	2	2.2%
CXR other	11	1.9%			
**Other TB risk factors**			**Other TB risk factors**		
TB contact	84	14.4%	TB contact	17	18.7%
PLHIV	5	0.86%	PLHIV	2	2.2%
Diabetes	34	5.8%	Diabetes	10	11.0%

**Table 2:** Test results of individual and pooled Ultra testing in Cambodia.

Xpert MTB/RIF Ultra Result	Individual Results n = 584		Pooled Results n = 153	
**Negative (N)**	**493**	84.4%	80	52.3%
MTB with rifampicin resistance detected (RR)	3	0.5%	0	0%
MTB with no rifampicin resistance detected (T)	74	12.7%	59	38.6%
MTB with low rifampicin resistance detected (Ti)	1	0.2%	3	1.9%
MTB trace detected (TT)	13	2.2%	11	7.2%

MTB = *Mycobacterium tuberculosis*.

**Table 3:** Pooled and individual results *.

Results from Pooled Testing		Results from Individual Testing		
		Total	MTB-Negative	MTB-Positive
**All pooled specimens**	153	584	493 (84.4%)	91 (15.6%)
MTB-Negative	80 (52.3%)	304	304 (100.0%)	0 (0.0%)
MTB-Positive	73 (47.7%)	280	189 (67.5%)	91 (32.5%)
RIF-Resistant MTB	0 (0.0%)	-	-	3 (3.3%)
Pooled specimens with all CXR abnormal individuals	66	253	175 (69.2%)	78 (30.8%)
MTB-Negative	6 (9.1%)	22	22 (4.5%)	0 (0.0%)
MTB-Positive	60 (90.9%)	231	153 (31.0%)	78 (85.7%)
RIF-Resistant MTB	0 (0.0%)	-	-	3 (3.3%)
Pooled specimens with mixed CXR results	17	64	55 (85.9%)	9 (14.1%)
MTB-Negative	6 (35.3%)	23	23 (4.7%)	0 (0.0%)
MTB-Positive	11 (64.7%)	41	30 (6.1%)	11 (12.1%)
RIF-Resistant MTB	0 (0.0%)	-	-	0 (0.0%)
Pooled specimens with all CXR normal individuals	70	267	265 (99.3%)	2 (0.7%)
MTB-Negative	68 (97.1%)	259	259 (52.5%)	0 (0.0%)
MTB-Positive	2 (2.9%)	8	6 (1.2%)	2 (2.2%)
RIF-Resistant MTB	0 (0.0%)	-	-	0 (0.0%)

MTB = *Mycobacterium tuberculosis*, CXR = chest X-ray, RIF = Rifampicin. * For individual results based on CXR group and MTB-negative or MTB-positive pooled sample percentages refer to the proportion of the total positive or negative results.

**Table 4:** Cost and time estimates utilizing different testing approaches.

	Total Cartridges Used	Cartridges Saved (%)	Total Time Required (Hours)	Time Saved (Hours)	Total Cost	Costs Saved (%)
Individual approach	584	-	876		USD 6050	
Pooled approach	433	151 (27)	650	226	USD 4485	1565 (27)
Hybrid approach *	382	202 (35)	573	300	USD 3958	2092 (35)

* Hybrid approach includes pooling only those with normal CXR readings and individually testing those with CXR abnormalities.
